# Supplementary material for: Deep learning for genomics using Janggu
Source: Nat Commun. 2020 Jul 13;11:3488. doi: 10.1038/s41467-020-17155-y (PMC7359359; doi:10.1038/s41467-020-17155-y)
Supplement: Supplementary file 1 — Supplementary Information [file 41467_2020_17155_MOESM1_ESM.pdf]

Deep learning for genomics applications with Janggu

Kopp *et al.*

Supplementary Table 1: DNA model for JunD prediction. DnaConv2D constitutes a wrapper that allows to scan both DNA strands with the same kernels.

Conv2D(10, (11, 1), 'relu')
DnaConv2D()
MaxPool2D(30, 1)
BatchNormalization()
Conv2D(8, (3, 1), 'relu')
GlobalMaxPooling()
BatchNormalization()
Dense(1, 'sigmoid')

Supplementary Table 2: DNase model for JunD prediction.

Conv2D(10, (5, 2), 'relu')
MaxPool2D(2, 1)
BatchNormalization()
Conv2D(5, (3, 1), 'relu')
GlobalMaxPooling()
BatchNormalization()
Dense(1, 'sigmoid')

Supplementary Table 3: DNA model for CAGE-tag prediction. λ was set to 0.0 and 0.2 for order one or higher order sequence features (two and three).

Dropout(λ)
Conv2D(10, (15, 1), 'relu')
MaxPool2D(5, 1)
BatchNormalization()
Conv2D(8, (5, 1), 'relu')
GlobalMaxPooling()
BatchNormalization()
Dense(1, 'linear')

Supplementary Table 4: Chromatin model for CAGE-tag prediction.

```
Concatenate()([dnase_signal, h3k4me3_signal])
BatchNormalization()
Dense(1, 'linear')
```

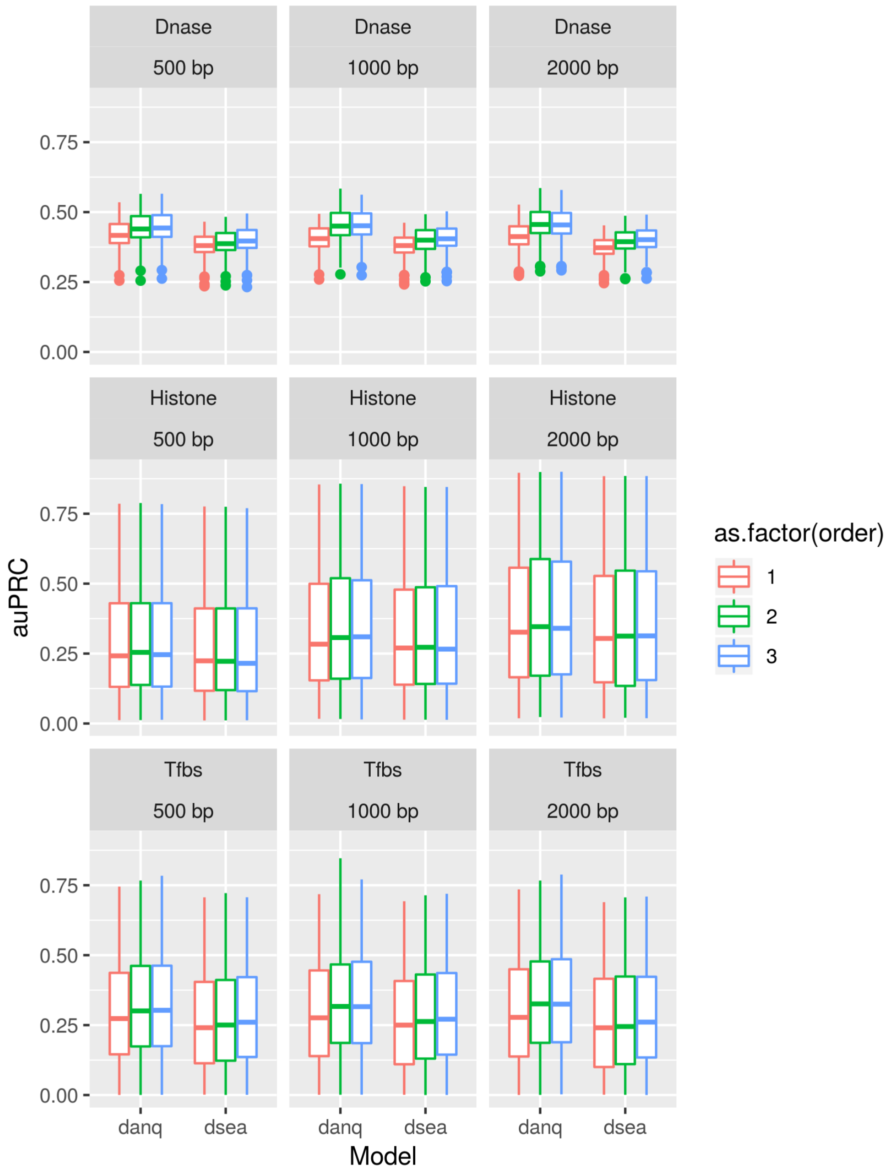

Supplementary Figure 1: **DeepSEA and DanQ comparison.** Performance comparison of DeepSEA and DanQ on the same benchmark data measured by the area under the precision-recall curve. The comparison dissects performances for different genomic features (Dnase, histone modifications and TF binding sites), different context window sizes (500bp, 1000bp and 2000bp) and different sequence encoding orders (order one, two and three).
